# Mechanical ventilation in acute brain injury patients with acute respiratory distress syndrome

**DOI:** 10.3389/fmed.2022.999885

**Published:** 2022-10-06

**Authors:** Mariyam Humayun, Lavienraj Premraj, Vishank Shah, Sung-Min Cho

**Affiliations:** ^1^Division of Neuroscience Critical Care, Department of Neurology, Neurosurgery, Surgery, Anesthesiology, and Critical Care Medicine, Johns Hopkins University School of Medicine, Baltimore, MD, United States; ^2^School of Medicine, Griffith University, Gold Coast, QLD, Australia; ^3^Critical Care Research Group, The Prince Charles Hospital, Brisbane, QLD, Australia

**Keywords:** ARDS, acute brain injury, mechanical ventilation, stroke, TBI-traumatic brain injury, ICP

## Abstract

Acute respiratory distress syndrome (ARDS) is commonly seen in patients with acute brain injury (ABI), with prevalence being as high as 35%. These patients often have additional risk factors for ARDS compared to general critical care patients. Lung injury in ABI occurs secondary to catecholamine surge and neuro-inflammatory processes. ARDS patients benefit from lung protective ventilation using low tidal volumes, permissive hypercapnia, high PEEP, and lower PO2 goals. These strategies can often be detrimental in ABI given the risk of brain hypoxia and elevation of intracranial pressure (ICP). While lung protective ventilation is not contraindicated in ABI, special consideration is warranted to make sure it does not interfere with neurological recovery. Permissive hypercapnia with low lung volumes can be utilized in patients without any ICP issues but those with ICP elevations can benefit from continuous ICP monitoring to personalize PCO2 goals. Hypoxia leads to poor outcomes in ABI, hence the ARDSnet protocol of lower PO2 target (55–80 mmHg) might not be the best practice in patients with concomitant ARDS and ABI. High-normal PO2 levels are reasonable in target in severe ABI with ARDS. Studies have shown that PEEP up to 12 mmHg does not cause significant elevations in ICP and is safe to use in ABI though mean arterial pressure, respiratory system compliance, and cerebral perfusion pressure should be closely monitored. Given most trials investigating therapeutics in ARDS have excluded ABI patients, focused research is needed in the field to advance the care of these patients using evidence-based medicine.

## Introduction

Acute Respiratory Distress Syndrome (ARDS) is described as a diffuse inflammatory process involving the pulmonary tissue, which is characterized by tissue edema, disruption of endothelial and epithelial linings in the lungs leading to hypoxemia and decreased compliance (1). It is defined by acute onset of pulmonary edema within a week of a clinical insult, development of bilateral radiographic opacities on chest imaging and a PaO2/FiO2 ratio of <300 mmHg in the setting of no overt cardiac etiology (1). The 2012 ARDS task force classified the disease based on the degree of hypoxemia, into mild (PaO_2_/FiO_2_ = 200–300 mmHg), moderate (PaO_2_/FiO_2_ = 100–200 mmHg), and severe (PaO_2_/FiO_2_ <100 mmHg) ARDS (1). In a large international cohort study (*n* = 29,144), period prevalence of ARDS in the intensive care unit (ICU) was 10.4% (2). The incidence varies geographically, with higher incidence in North America compared to Europe (3). ARDS is associated with significant ICU mortality of 35.3% and, among survivors, a high risk of long-term functional and cognitive impairment (2, 4).

## ARDS in acute brain injury: Epidemiology and risk factors

While ARDS has been well-described in general critical care populations, it remains poorly characterized in the neurocritical care population. Acute respiratory failure is the most common extra-cranial complication among patients with acute brain injury (ABI) (5), who often require mechanical ventilation due to poor mental status (Glasgow Coma Scale ≤8) (6), inability to protect airway, given loss of brainstem reflexes and intracranial pressure (ICP) elevation. A single center prospective observational study evaluating ARDS/Acute Lung Injury (ALI) in the neurocritical care population reported an incidence of 35% (7). This was higher compared to prior studies where occurrence varied from 10 to 31% (8–10).

Pneumonia and extra-pulmonary sepsis are the most common risk factors for development of ARDS (11). Other less common risk factors include aspiration, trauma, and blood transfusions amongst many others (11). Patients with ABI often have additional predispositions. Hoesch et al. described a strong association between loss of cough or gag reflexes and development of ARDS in patients with ABI. The absence of these reflexes represents an inability to protect airway, whereby increasing the risk of aspiration. In addition, the loss of lower brain stem function can reflect the severity of ABI, which may independently play a role in acute lung injury (7). Use of vasoactive agents, history of drug abuse and hypertension have also been associated with development of ARDS in ABI (12).

Existing studies evaluating ARDS in acute brain injury (ABI) have largely focused on traumatic brain injury (TBI) and acute stroke patients.

### TBI and ARDS

TBI patients are prone to developing ARDS as a consequence of polytrauma, leading to pulmonary contusions and need for massive transfusion of blood-products in the setting of hemorrhagic shock. Prevalence of ARDS in TBI appears to have increased from 2% in 1988 to 22% in 2008, partly related to improved diagnostic definition (13). A systematic review of 2,830 patients showed that the pooled prevalence of ARDS was 19%, of which 25% had severe ARDS (14). As expected, TBI patients developing ARDS have higher mortality (43 vs. 33%, *p* = 0.01) and lower proportion of good functional outcome defined by Glasgow Outcome Scale ≥ 4 (23 vs. 34% *p* = 0.02), compared to those without ARDS (14).

ARDS in TBI was seen more commonly in young males, white race, and patients with existing hypertension (13). In hospital complications including sepsis and cardiovascular dysfunction were independent predictors for ARDS (13). Severity of ABI, defined by GCS and radiographic findings, has also been associated with the development of ARDS (15).

### Stroke and ARDS

The prevalence of ARDS varies in different types of stroke. ARDS after ischemic stroke is a rare entity in the United States; a large retrospective study of ischemic stroke patients documented the prevalence of ARDS to be only 4% (16). For ICH patients who required mechanical ventilation, Elmer et al. reported the prevalence of ARDS to be 27% (*n* = 697). They also found that use of high tidal volume (>8 ml/kg) was strongly associated with development of ARDS in this subset (Hazard Ratio = 1.74, 95% Confidence Interval=1.08–2.81) (17). In a Chinese cohort of stroke patients, 4% (*n* = 1,495) developed aspiration related ARDS, 70% of which presented with an ischemic stroke, 26% with intracerebral hemorrhage (ICH) and 4% with subarachnoid hemorrhage (SAH) (18).

A recent meta-analysis investigating ARDS in aneurysmal SAH patients reported that the overall prevalence of ARDS was 15% while the prevalence for moderate/severe ARDS was 13%. SAH with ARDS had a significantly lower survival rate compared to those without ARDS (49 vs. 79% *p* = 0.028) (19). A nationwide SAH database from the United States showed that the incidence of ARDS in 2008 was 37.6% increased by 2% compared to 1993 (20). Mazeraud et al. reported a lower incidence of ARDS (3.6%) in their cohort of aneurysmal SAH patients from Europe (21). ARDS was associated with worse neurological outcomes even after adjustment for various confounding factors such as initial severity of neurological injury and duration of mechanical ventilation (21). Male sex, pneumonia, and high grade SAH (Hunt and Hess ≥3) have been associated with ARDS in aneurysmal SAH (19).

## Pathophysiology of lung injury after ABI: The brain-lung conflict

The lungs and the brain are intimately connected and injury in one often perpetuates injury to the other. Multiple hypotheses exist for the mechanism of lung injury secondary to ABI. ABI leads to a systemic pro-inflammatory state. Cerebral edema causes local release of inflammatory mediators, which then escape into systemic circulation via the disrupted blood-brain barrier. Activation of inflammatory cascades and cytokine release can cause pulmonary tissue damage (12, 22).

Elevated ICP activates the sympathetic nervous system. Catecholamines are released into the blood stream, leading to pulmonary vasoconstriction and an inflammatory surge, which can be responsible for endothelial damage and vascular leakage (22). This blast theory is also believed to be the cause for neurogenic pulmonary edema which can often present with acute respiratory distress similar to ARDS (22). Impairment of the parasympathetic nervous system, including the immunoregulatory function of the vagus nerve and loss of cholinergic anti-inflammatory pathways can also predispose to lung injury in ABI patients (22). ABI can lead to increased respiratory system elastance, flow resistance and extravascular lung water, all of which can increase the risk to develop ARDS (22).

A second hit phenomenon has also been described, where secondary processes like mechanical ventilation, superimposed infections and surgeries may further damage pulmonary tissue, increasing chances of developing ARDS (22).

## Mechanical ventilation strategies for ARDS

The primary strategy for mechanical ventilation in ARDS is to correct hypoxemia, while preventing the perpetuation of lung injury mediated by positive-pressure ventilation or ventilator-induced lung injury. This is known as lung protective ventilation (LPV) and involves the use of a controlled ventilator mode with low tidal volumes to prevent volutrauma, positive end expiratory pressure (PEEP) to correct hypoxemia and prevent atelectrauma, all while maintain lower airway pressures to prevent barotrauma (23).

The landmark ARDSnet trial, randomized 861 patients to receive low tidal volume (4–6 ml/kg) vs. standard (10–12 ml/kg) ventilation strategy (23). Tidal volumes were lowered to maintain plateau pressures (static airway pressures) of <30 cm H_2_O. The trial demonstrated a significant survival benefit among the low tidal volume group (23). Lower lung volumes prevent volutrauma by preventing overinflation of aerated alveoli, and maintaining plateau pressures <30 cm H_2_O helps prevent barotrauma. As expected, however, lower tidal volumes are associated with higher arterial PCO_2_ and lower pH (23), an undesired consequence in ABI, as discussed below.

Higher PEEP is used to recruit more alveoli and improve oxygenation by reducing pulmonary shunting. In addition, PEEP prevents alveolar collapse during end-expiration, reducing atelectrauma. However, Brower et al. demonstrated that clinical outcomes were similar with low or high PEEP levels, provided a low tidal volume strategy and plateau pressure of <30 cm H_2_O was maintained (24). Driving pressure, the pressure generated during alveolar opening, is another important physiological parameter that can be used to titrate the tidal volume taking into account the respiratory system compliance (25). This was first described in a retrospective observational study, which reported that maintaining lower driving pressures was associated with improved mortality in ARDS (25).

Finally, recruitment maneuvers, involving very high PEEP levels for brief durations to recruit more alevoli, have been shown to have minimal outcome benefit in clinical trials (24, 26), with only a brief increase in oxygen saturation and concurrent transient hypotension (24).

## Mechanical ventilation in ARDS after ABI

Mechanical ventilation is frequently necessary in patients with critical brain injury. Although invasive mechanical ventilation can be a life-saving measure for acute respiratory failure, this modality may have harmful effects on the brain. As most clinical trials investigating mechanical ventilation in ARDS have excluded neurocritical care population, there is no robust data available to guide clinicians on how to manage ARDS after ABI in the neurocritical care unit. Majority of ARDSnet clinical trials excluded patients with increased ICP or neuromuscular weakness (23).

Traditional LPV strategy for ARDS can be challenging in brain injured patients. While ICP monitoring and control is a crucial therapeutic target in many of these patients, some of the LPV strategies have a potential of increasing ICP.

### Tidal volume, hyperventilation, and PCO_2_

**Figure 1:**
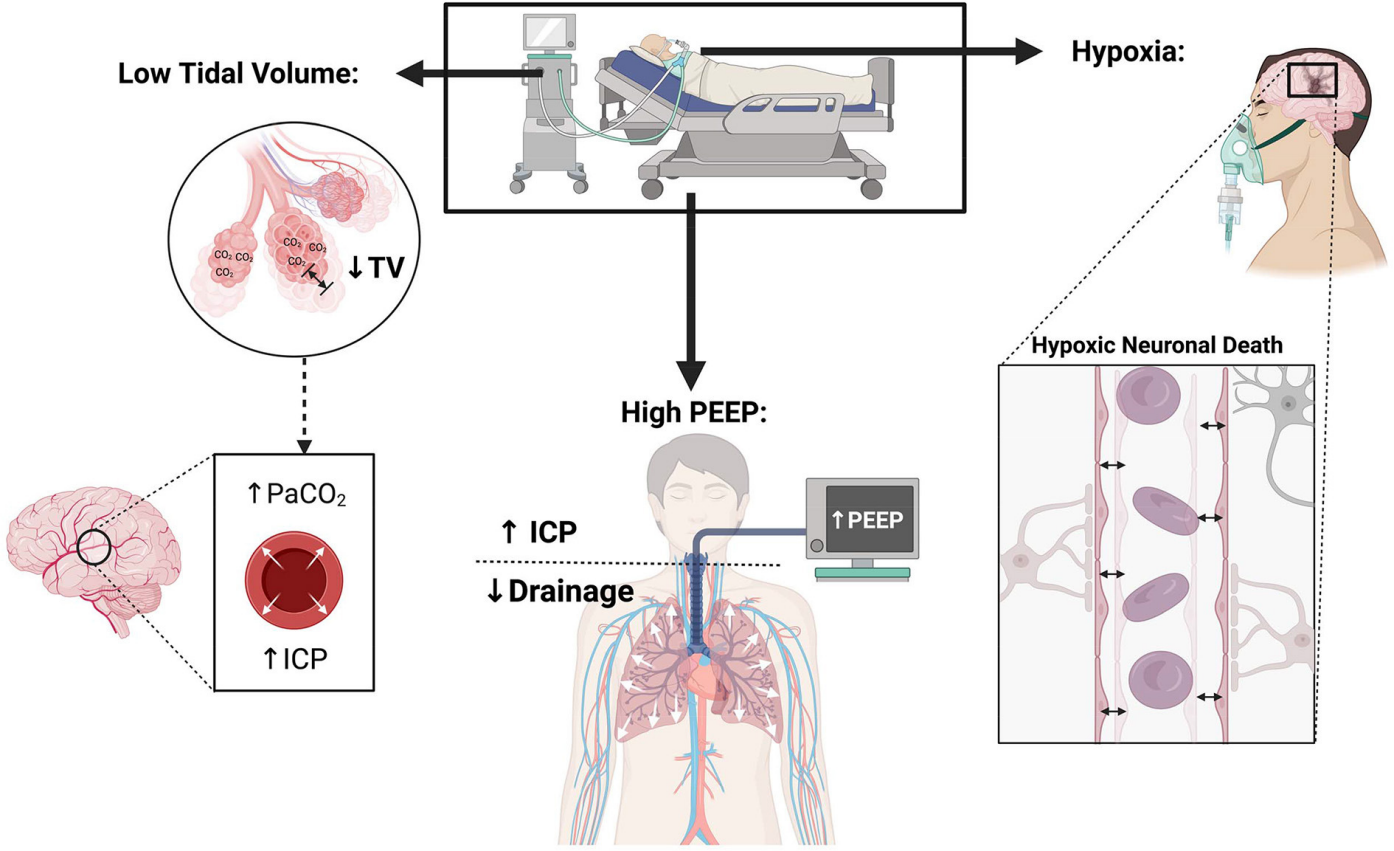
Physiological consequences of lung protective ventilation in patients with neurological injury.

A lower tidal volume (6 ml/kg) for ARDS patients leads to hypercarbia and acidosis. Guidelines recommend goal pH of 7.30–7.45, while allowing permissive hypercapnia, in ARDS patients. Hypercarbia causes cerebral vasodilatation, which can theoretically lead to ICP elevation (Figure 1).

A retrospective analysis of 12 SAH patients (Hunt and Hess grade 3–5) with ARDS evaluated ICP in the setting of permissive hypercapnia. Intraparenchymal monitors were placed to measure ICP and mean oxygen pressure. When TV of 6–8 ml/kg and PEEP of 10–15 mmHg were implemented, there was no ICP elevation (2–18 mmHg) in patients despite PCO_2_ ranging between 50 and 60 mmHg (27).

Patients with TBI are typically ventilated to achieve normal to low PCO_2_ levels (28). Hyperventilation can be used as one of the strategies to decrease ICP if needed. Brain Trauma Foundation guidelines advise against use of prolonged hyperventilation with PCO2 targets of ≤25 mmHg to prevent ICP elevation. The first 24 h are crucial for cerebral perfusion so effort should be made to avoid hyperventilation. In cases when it is utilized, they recommended using a cerebral oxygenation measure when possible (Brain tissue oxygen partial pressure, BtpO_2_ or jugular venous oxygen saturation, SjO_2_) (29). An international observational study of 86 ABI patients showed that higher tidal volumes (9.5 vs. 10.4 ml/kg, *p* = 0.001) and hyperventilation (12.7 vs. 14.2 *p* = 0.0006) were associated with development of acute lung injury (30).

Torre et al. in their review article, addressing management of ARDS in TBI patients, concluded that LPV is not an absolute contraindication in neurological patients (28). Individualized approach using ICP monitors may be needed in patients to set PCO_2_ goals in cases of elevated ICP and ARDS (31).

The European Society of Intensive Care Medicine Consensus recommends using normal targets (35–45 mmHg) for PCO_2_ in ABI patients who do not have ICP elevation and ARDS. In brain injured patients, who do not have significant ICP elevations but have concomitant ARDS, LPV is recommended. The guideline was unable to make any specific recommendations for patients with ICP crisis and concurrent ARDS (6). However, it is important to note that these recommendations are based on limited high-quality data.

### Oxygenation

Hypoxia can be detrimental in neurocritical care patients where adequate oxygen delivery is critical for recovery; attention should be paid to maintain normal partial pressures of arterial oxygen. A guideline from the European Society of Intensive Care Medicine recommended maintaining arterial oxygen levels of 80–120 mmHg in brain injured patients (6), which contrasts with ARDSnet practice of oxygenation goals of 55–80 mmHg (23) that allows relative mild hypoxia (Figure 1). Torre et al. in their review of TBI patients with ARDS emphasized the need for normoxia and avoiding hypoxia in TBI patients (28). Hyperoxia (>200 mmHg) is also associated with higher mortality and worse short-term functional outcomes in TBI (32). A recent review article addressing management of ARDS in severe ABI also recommended higher PO2 targets of >110 mmHg if allowed from a lung compliance perspective (31). Thus, for ABI patients with ARDS, it may be reasonable to use a higher PO_2_ goal than what has been recommended in ARDS patients without ABI (55–80 mmHg) (6, 28, 31). Oxygen targets may be modified in settings where brain tissue oxygen (PbtO_2_) monitoring is available, understanding that there is no high-quality data on the outcome benefit of PbtO_2_ in patients with ICP crisis.

### PEEP

Increasing PEEP elevates the intrathoracic pressure, causing a decrease in the cerebral venous return, which may subsequently increase ICP (31) (Figure 1).Given this possible effect on ICP, use of high PEEP and recruitment maneuvers is contentious in the neurocritical care field (28).

A small sample of acute stroke patients showed that PEEP up to 12 mmHg did not significantly elevate ICP, however, was associated with decrease in cerebral perfusion pressure (CPP) in patients with impaired cerebral autoregulation, where a linear relationship exists between systemic mean arterial pressure (MAP) and CPP. If MAP can be effectively adjusted to maintain CPP, higher PEEPs might be safe to use (33). Zhang et al. showed that among 9 severe brain injury patients, high PEEP (up to 21 mmHg) negatively impacted ICP and CPP. They reported a significant correlation between PEEP and ICP (*R* = 0.637, *p* < 0.05), and PEEP and CPP (*R* = −0.584, *p* < 0.05) (34). However, the relationship between PEEP and ICP may depend on lung compliance, ARDS severity, and brain compliance based on severity of ABI.

PEEP leads to alveolar recruitment but can also cause alveolar hyperinflation, particularly in preserved areas of the lungs. Alveolar hyperinflation leads to an increase in the arterial carbon dioxide concentration which can elevate ICP (35). Thus, lung compliance can also determine the effect of PEEP on the intracranial system. Caricato et al. in their prospective study of 21 ABI patients, compared effects of PEEP in patients with low (<45 ml/cm H_2_O) vs. normal lung compliance. They showed those with lower respiratory system compliance did not have significant changes in cerebral and system hemodynamics with increases in PEEP. They suggested monitoring lung compliance in ABI patients who require higher PEEP for treatment of hypoxemia (36). Thus, PEEP up to 12 cm of H_2_O may be safe and higher PEEP levels can be used to improve oxygenation in ARDS, provided other parameters including MAP, CPP and respiratory system compliance can be optimized (22, 31).

In summary, there is limited evidence to guide mechanical ventilation strategies in ABI patients who develop ARDS. At this moment, close monitoring of ICP and brain tissue oxygen, if available, while adjusting mechanical ventilation is recommended, particularly in severe ABI patients who are high risk of progressive neurologic injury. The VENTIBrain study, is an ongoing international multicenter prospective observational study looking into practice of mechanical ventilation in brain injured patients and will likely provide valuable insight into current practices and outcomes of this population (37). Continued research is required in the field to further advance the care in this field.

## Other ARDS therapeutics

Adjunctive therapy is used in severe ARDS includes prone ventilation, neuromuscular blockade, and inhaled nitrous oxide. Each practice needs to be evaluated carefully when used in the neurocritical care setting for ARDS after ABI.

The PROSEVA trial showed that prone positioning in severe ARDS significantly improved mortality, however excluded patients with severe ABI (38). Prone-ventilation helps relieve posterior lung compression and improves aeration and blood-flow to the posterior alveoli, significantly reducing pulmonary shunting, and consequently improving oxygenation. However, proning can lead to an elevation in ICP, particularly among patients with severe ABI (39). In a small retrospective study of 27 patients with ICP monitoring requiring prone ventilation for ARDS, 52% developed intracranial hypertension and all these patients had an ICP > 17.5 mm Hg prior to prone positioning (40). Thus, the benefit of prone position should be weighed against the risk of ICP crisis based on patient's severity of neurological and respiratory injury and ICP monitoring should be considered during prone ventilation, particularly given loss of neurological assessments.

Sedation and paralytics are often used for ventilator desynchrony and to improve oxygenation. Although the use of these agents can obscure meaningful neurological examinations, they can be used to control ICP for refractory ICP crisis. Inhaled nitric oxide can also be used to improve oxygenation. Some preliminary data suggested that nitric oxide can be helpful in improving cerebral autoregulation though more research in human subjects is necessary (41). Extracorporeal membrane oxygenation (ECMO), mostly veno-venous, is recommended for medically refractory ARDS as a rescue therapy. Despite the challenges in maintaining ECMO support in patients with ABI, especially ICH, as VV-ECMO can be maintained without anticoagulation, careful multidisciplinary discussion should be pursued to offer this rescue therapy to select patients who may have a chance of both lung and brain recovery.

## Conclusion

Although ARDS in neurocritical care population is not infrequent, the data and clinical guidance on management strategy of ARDS after ABI is scarce. Lung protective ventilation, which uses lower tidal volumes, high PEEP, and permissive hypercapnia, is viewed with caution in brain injured patients given the potential to increase ICP and worsening neurological outcome. However, LPV should not be a contraindication in ARDS after ABI and should be pursued, with close monitoring of neurophysiological parameters, such as ICP, CPP, brain tissue oxygen and systemic parameters such as MAP, arterial PCO_2_ and PO_2_. Further research is needed to demonstrate the safety and efficacy of different ARDS management strategies in the neurocritical care population.

## Author contributions

MH and S-MC: study concept and design and first drafting of the manuscript. LP: figure. All authors are critical revision for important intellectual content and final approval of the manuscript.

## Funding

S-MC is supported by the National Heart, Lung, and Blood Institute (NHLBI 1K23HL157610).

## Conflict of interest

The authors declare that the research was conducted in the absence of any commercial or financial relationships that could be construed as a potential conflict of interest.

## Publisher's note

All claims expressed in this article are solely those of the authors and do not necessarily represent those of their affiliated organizations, or those of the publisher, the editors and the reviewers. Any product that may be evaluated in this article, or claim that may be made by its manufacturer, is not guaranteed or endorsed by the publisher.
